# Surgical emergencies confounded by H1N1 influenza infection - a plea for concern

**DOI:** 10.1186/1749-7922-5-6

**Published:** 2010-03-01

**Authors:** Benjamin Person, Hany Bahouth, Eran Brauner, Offir Ben-Ishay, Amitai Bickel, Yoram S Kluger

**Affiliations:** 1 Department of General and Acute Care Surgery, Rambam Healthcare Campus, POB 9602, Haifa, 31096, Israel; 2 Department of Surgery, West Galilee Hospital, POB 21, Nahariya, 22100, Israel

## Abstract

The outbreak of the H1N1 influenza pandemic resulted in unprecedented, overwhelming exposure in the medical and lay media, with the obvious focus of healthcare providers being on patients in internal medicine or intensive care settings.

Recently, we treated 3 patients with various surgical emergencies who were also diagnosed with active H1N1 influenza. The purpose of this report is to bring the issue of H1N1 flu in association with surgical emergencies to the forefront of the literature, and suggest that surgical diseases might be significantly accentuated in patients with H1N1 influenza.

## Background

Since the outbreak of the H1N1 influenza pandemic in April 2009, an enormous body of literature presented various aspects of this new disease. Most of the reports describe epidemiological characteristics [1,2] or the medical course and outcomes of patients with H1N1 [3-5], and are therefore presented mostly in the internal medicine or critical care medicine literature [6-9].

Recently, our acute care surgery service was confronted with 3 patients who presented with relatively common surgical emergencies; however, due to concurrent H1N1 infection, their hospital course was unexpectedly and dramatically extraordinary.

### Case 1

**Figure 1:**
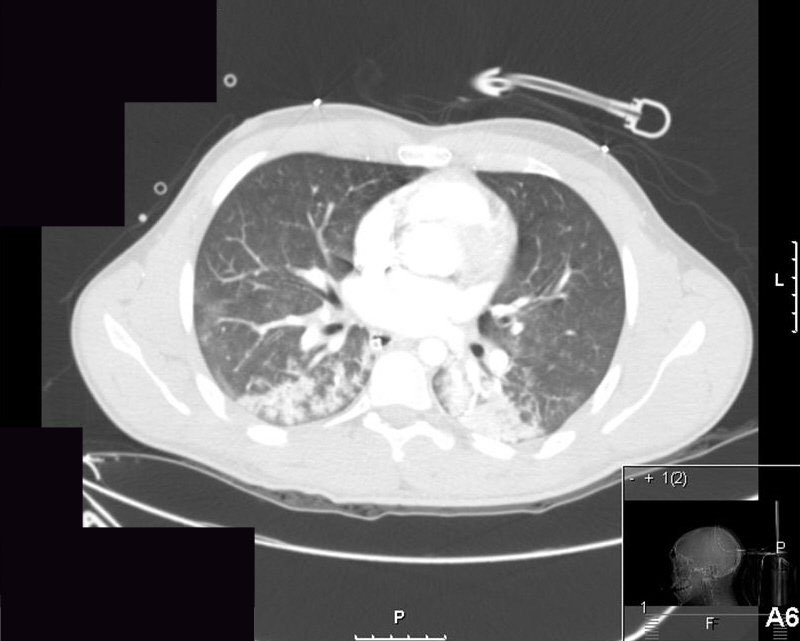
**CT scan of the chest showing bilateral, bibasilar infiltrates**.

A healthy 19-year-old man fell from a 3-meter-long ladder and hit his head. At the scene he was comatose with a Glasgow Coma Score of 4; a right dilated and unresponsive pupil and no other obvious injuries were identified. He was intubated, ventilated and transferred to our trauma center. His family members reported that he complained of having a sore throat in the preceding 2 days. On admission, the initial significant physical findings were a fever of 39.5°C, a heart rate of 150 beats/min and normal blood pressure. A large right fronto-parietal subcutaneous hematoma and a dilated right pupil were revealed. The chest X-ray was consistent with bilateral infiltrates that were presumed to be lung contusions or the result of aspiration. An abdominal ultrasound did not show intra-peritoneal, pelvic or pericardial fluid. A CT scan of the brain revealed a large fronto-parietal epidural hematoma on the right with a significant mass effect, and multiple fractures of the frontal and temporal bones. A CT scan of the abdomen and pelvis was normal, and a CT scan of the chest showed the same bilateral, bibasilar infiltrates that were seen on the initial chest X-ray (figure 1). The patient underwent an emergency craniotomy with evacuation of the epidural hematoma and insertion of an intracranial pressure monitoring catheter (ICP). During the operation, due to a significant yet unexplained decrease in the blood pressure the patient underwent an intraoperative trans-esophageal echocardiography that demonstrated a severe global left ventricular dysfunction with an ejection fraction of 15%. At that point the differential diagnosis was either of acute myocarditis related to a suspected streptococcal throat infection, cardiac contusion or catecholamine induced cardiomyopathy [10]. The patient was transferred to the intensive care unit (ICU); he was sedated, pharmacologically paralyzed, mechanically ventilated and required large doses of vasopressors to maintain a normal blood pressure. The initial Creatine Kinase (CK) level was 1800 U/L (n = 30-180) and Troponin I level was 56 ng/ml (n = 0-0.2). On the next day his temperature was 40.7°C, heart rate 156 beats/min and blood pressure 113/61 mmHg; he was diagnosed with acute respiratory distress syndrome (ARDS), acute renal failure, rhabdomyolysis with repeat CK levels of 12516 U/L and urinary myoglobin levels 936000 μg/L (n = up to 1000). Subsequently, the patient did not regain consciousness despite complete cessation of sedative and paralytic agents and gradually but very quickly entered a state of multi organ failure (MOF). The diagnosis of H1N1 influenza was made 2 days after his admission by real time PCR testing, and he received intravenous immunoglobulin (IVIG) and Oseltamivir. Despite aggressive attempts of resuscitation, the patient died 7 days from admission with a final diagnosis of viral myocarditis and pneumonitis related to H1N1 influenza.

### Case 2

A 29-year-old female patient who was 29 weeks pregnant presented to another hospital complaining of shortness of breath, fever and epigastric pain. Her past history was remarkable for a caustic esophageal injury that was treated by esophago-gastrectomy and colonic interposition 8 years ago. Soon after her admission she went into a state of severe respiratory distress, was intubated and mechanically ventilated. A CT scan of the abdomen showed a dilated large bowel that was presumed to be related to a left-lower-lobe pneumonia. She was transferred to our hospital for further treatment. On admission the patient was sedated, mechanically ventilated, oliguric, tachycardic to 160 beats/min, hypotensive with a systolic pressure of 70 mmHg and had profound lactic acidosis. Due to severe fetal distress she was transferred to the operating room for emergency cesarean section. A 1,100 gram male fetus was delivered, intubated, ventilated and after stabilization was transferred to the neonatal intensive care unit (NICU). On exploration of the abdominal cavity, the patient's almost entire remaining colon and 130 cm of distal small bowel were necrotic as a result of an adhesion from the previous surgery that caused complete bowel obstruction. The necrotic bowel was resected and the ends stapled off without an anastomosis or a stoma. This was elected due to hemodynamic instability. The abdomen was temporarily closed and a planed second-look laparotomy to determine the fate of the remaining bowel was scheduled. The patient was transferred to the ICU for further stabilization. On the next day, 30 hours after the first operation, the patient underwent a second-look laparotomy. Surprisingly, an additional segment of 150 cm of distal small bowel was necrotic and was therefore resected. The patient remained with approximately 120 cm of jejunum, and even this segment looked somewhat pale and non-viable. Again, the abdomen was temporarily closed for a planned third laparotomy. At that point the patient was diagnosed as being positive for H1N1 influenza by real time PCR test, and began receiving appropriate treatment. On the next day she underwent another laparotomy during which and additional segment of 40 cm of distal jejunum was resected, and an end-stoma was fashioned. Gradually she recovered in the ICU, and was transferred to a general surgical ward one week after admission to the hospital. She now has approximately 80 cm of normal small bowel ending in a stoma, and is getting her nutritional support by total parenteral nutrition (TPN). Repeat testing for H1N1 was negative one week after the first positive result.

### Case 3

**Figure 2:**
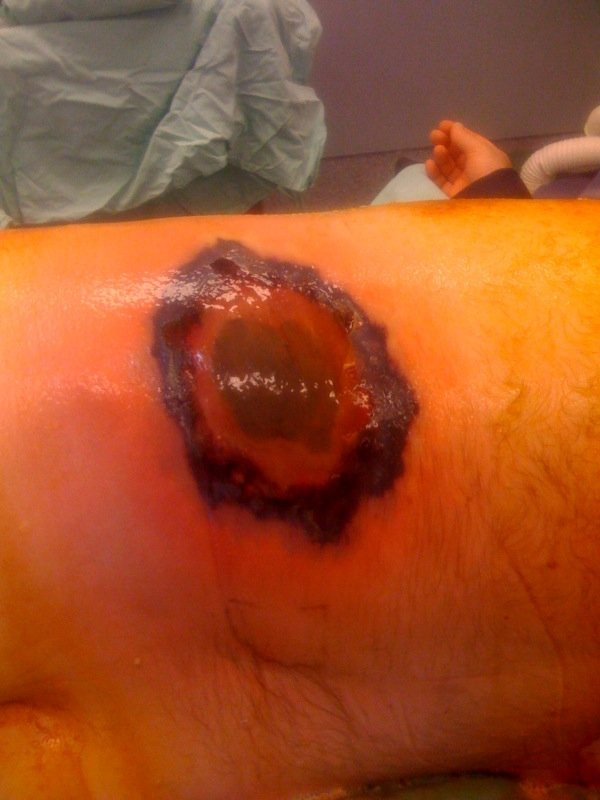
**The lesion on the patient's left flank before the first operation**.

**Figure 3:**
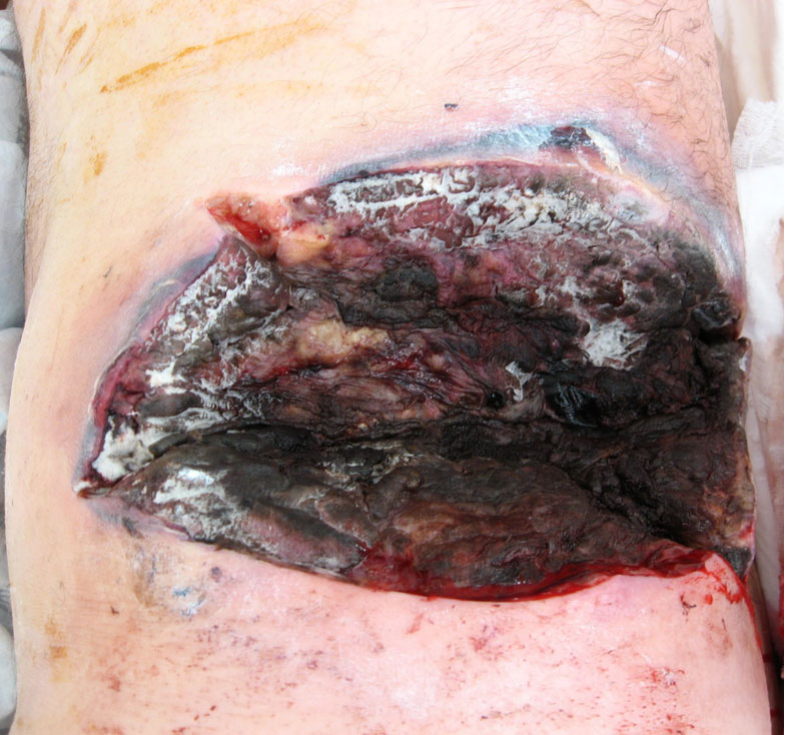
**Surgical wound of the patient's left flank showing necrotizing soft tissue infection covered by white patches of fungi**.

A 59-year-old male patient with diabetes mellitus type 2 treated with oral agents, chronic obstructive pulmonary disease (COPD) treated with inhalers and oral steroids, and hyperlipidemia treated with statins was admitted to an internal medical ward 2 weeks prior due to H1N1 associated pneumonia. He was treated with Oseltamivir and discharged after 2 days in the hospital. He was hospitalized again several days later due to continuous symptoms of acute upper respiratory infection. He received symptomatic treatment for several days. During this admission, the staff noted a lesion in his left flank (figure 2). He underwent an emergency operation for debridement of a suspected necrotizing soft tissue infection in another hospital. The next day he was operated again due to expansion of the necrosis, and treated with broad spectrum antibiotics. Because of rapid deterioration and septic shock he was transferred to our medical center for hyperbaric Oxygen therapy (HBO). Histopathology results from the necrotic lesion revealed an infection with Mucormycosis and the patient was put on intravenous Amphotericin B therapy. A test for H1N1 influenza was again positive nearly 3 weeks following his previous positive test, and treatment with Oseltamivir was restarted. He underwent 2 more extensive debridements of his left flank (figure 3) and subsequently an extensive debridement of both his thighs and left arm due to disseminated Mucormycosis infection. The patient expired 4 days after his admission due to septic shock and MOF.

## Discussion

The first case reported here is a relatively straightforward trauma scenario encountered by acute care surgeons on a nearly daily basis. The reported outcomes of patients with epidural hematomas who undergo early operative intervention is usually good to reasonable [11], especially in young and healthy patients. Our patient probably had H1N1 influenza for several days prior to falling from the ladder; possibly, being ill was the reason he fell in the first place. We speculate that had the patient been in perfect health while being injured, his hospital course and outcome may have been totally different.

H1N1 influenza in pregnancy has been discussed in the literature before [12-15]. The patient described in the second case report had a remarkable past history for having a total gastro-esophagectomy and colonic interposition due to caustic injury 8 years ago. She had one vaginal delivery 6 years ago, and had a relatively normal life since then. The complete bowel obstruction she had went un-noticed in the first hospital due to confounding findings of left-lower-lobe pneumonia and severe respiratory distress. The emergency cesarean section revealed the true magnitude of the catastrophic consequences of the adhesions from her previous operation. The surprising findings were the progress and extent of the small bowel necrosis that was seen on the subsequent laparotomies. The expected course of bowel necrosis following complete obstruction is that after resection of the necrotic segment and adhesiolysis, the remaining bowel either recovers or demarcates and demonstrates the clear border between normal and necrotic bowel. In our patient, 150 cm of small intestine that looked relatively normal during the first operation were found necrotic 30 hours later, and an additional segment of 40 cm was further resected in the third operation. This progressive ischemia/necrosis may be attributed to the state of septic shock the patient was in, largely caused by H1N1 influenza infection. Fortunately, the patient recovered albeit with a short bowel and permanent TPN therapy.

The third case is slightly more complicated due to baseline poor medical condition of the patient. This is a patient with uncontrolled diabetes, hyperlipidemia, hypertension and COPD treated with steroids that also had H1N1 influenza. Mucormycosis infection in immune compromised patients is a well known entity [16,17]. This diabetic patient was also treated with steroids for severe COPD, and spent a long time in a hospital due to resistant H1N1 infection. He developed a cutaneous Mucormycosis infection that very quickly disseminated in spite of maximal appropriate therapy and resulted in the patient's demise.

In this era the medical and lay literature is flooded with information about the H1N1 influenza; however, due to the nature of their practice, surgeons encounter this disease less frequently, and are less minded to its potential hazards. The purpose of this short report is to highlight the possible association of H1N1 influenza outbreak with surgical emergencies and demonstrate a possible poor outcome of surgical patients who contract H1N1 influenza. We speculate that concurrent infection with H1N1 influenza with relatively common surgical entities may aggravate the patients' course and potentially play a major role in their final outcome.

## Competing interests

The authors declare that they have no competing interests.

## Authors' contributions

BP - conceived of the study, and participated in its design and coordination and drafted the manuscriptHB - participated in data acquisition and drafting of the manuscript

EB - participated in data acquisition and drafting of the manuscript

OBI - participated in data acquisition and drafting of the manuscript

AB - participated in data acquisition and drafting of the manuscript

YK - conceived of the study, and participated in its design and coordination

All authors read and approved the final manuscript

## Consent

Since two of the patients described in this paper expired, written informed consent was not obtained from them for publication of this case report and accompanying images. The third patient, who is still alive, could not give a written consent due to her general condition until the date of the publication of this paper. None of the images presented in this paper are of this patient.
